# Lactate exacerbates lung damage induced by nanomicroplastic through the gut microbiota–HIF1a/PTBP1 pathway

**DOI:** 10.1038/s12276-023-01129-3

**Published:** 2023-12-01

**Authors:** Lihui Xuan, Zheng Xu, Jinhua Luo, Yin Wang, Yuhui Yan, Can Qu, Zuozhong Xie, Magdalena Skonieczna, Ping-Kun Zhou, Ruixue Huang

**Affiliations:** 1 Department of Occupational and Environmental Health, Xiangya School of Public Health, Central South University, Changsha, Hunan Province 410078 China; 2 Translational Medicine Research Center, Naval Medical University, 800, Xiangyin Road, 200433 Shanghai, People’s Republic of China; 3 Department of Otolaryngology Head and Neck Surgery, The Second Xiangya Hospital, Central South University, Changsha, 410011 Hunan Province China; 4 Department of Systems Biology and Engineering, Silesian University of Technology, Institute of Automatic Control, Akademicka 16, Gliwice, 44-100 Poland; 5 Biotechnology Centre, Silesian University of Technology, Krzywoustego 8, Gliwice, 44-100 Poland; 6 Department of Radiation Biology, Beijing Key Laboratory for Radiobiology, Beijing Institute of Radiation Medicine, Beijing, 100850 China

**Keywords:** Chemokines, Cancer prevention

## Abstract

Exposure to nanomicroplastics (nano-MPs) can induce lung damage. The gut microbiota is a critical modulator of the gut–lung axis. However, the mechanisms underlying these interactions have not been elucidated. This study explored the role of lactate, a key metabolite of the microbiota, in the development of lung damage induced by nano-MPs (LDMP). After 28 days of exposure to nano-MPs (50–100 nm), mice mainly exhibited damage to the lungs and intestinal mucosa and dysbiosis of the gut microbiota. Lactate accumulation was observed in the lungs, intestines and serum and was strongly associated with the imbalance in lactic acid bacteria in the gut. Furthermore, no lactate accumulation was observed in germ-free mice, while the depletion of the gut microbiota using a cocktail of antibiotics produced similar results, suggesting that lactate accumulation in the lungs may have been due to changes in the gut microbiota components. Mechanistically, elevated lactate triggers activation of the HIF1a/PTBP1 pathway, exacerbating nano-MP-induced lung damage through modulation of the epithelial–mesenchymal transition (EMT). Conversely, mice with conditional knockout of *Ptbp1* in the lungs (*Ptbp1*^flfl^) and *PTBP1*-knockout (*PTBP1*-KO) human bronchial epithelial (HBE) cells showed reversal of the effects of lactate through modulation of the HIF1a/PTBP1 signaling pathway. These findings indicate that lactate is a potential target for preventing and treating LDMP.

## Lactate: the hidden culprit in microplastic-induced lung damage

This study examined the effects of nano-microplastics (minuscule particles of plastic found in the environment) on human health. The impact of these particles on the human respiratory and gastrointestinal (related to the stomach and intestines) systems remains unknown. The scientists found that exposure to nano-microplastics can concurrently harm the lungs and intestines in mice. This harm was connected to an increase in lactate (a type of acid produced by the body) levels in these organs, causing an imbalance in gut bacteria (microorganisms that live in the digestive tract). The study also noted that this harm could be transmitted via gut bacteria and that lactate intensified the lung harm induced by the nano-microplastics. This research deepens our understanding of the potential health risks of nano-microplastics exposure. The results could aid in devising strategies to prevent and manage resultant health problems. This summary was initially drafted using artificial intelligence, then revised and fact-checked by the author.

## Introduction

Nanomicroplastics (nano-MPs) are widespread in the environment and are a threat to human health^1^. In Europe, 63,000 to 430,000 tons of MPs are released into the environment, while in North America, this is estimated to range from 44,000 to 300,000 tons due to agricultural activities^2^. Inhalation and ingestion are the two major entry routes of nano-MPs into the human body, leading to damage in the respiratory system and gastrointestinal (GI) tract^3^. Pulmonary toxicity occurs at concentrations of 98.4–196.79 µg/mL^4^, and dysregulation of the microbiota–brain axis has been reported in rats after exposure to low doses of nano-MPs (50 and 100 µg/L) for 24 weeks^5^. However, the effects of nano-MPs on the respiratory system and GI tract have not been fully elucidated.

There is accumulating evidence that the gut microbiota and derived metabolites or products are involved in lung diseases such as bronchiolitis, asthma^6^, and lung fibrosis^7^. Such interactions are evidence of a gut–lung axis^8^. Lactate formed from pyruvate allows the reoxidation of cytosolic NADH to maintain glycolysis^9^. Nevertheless, lactate formation is an important characteristic of the Warburg effect under anaerobic conditions^9^. Lactate is produced by classic lactate-producing bacteria such as *Lactobacillus*, *Carnobacterium*, and *Oenococcus* species^10^. It is a key metabolite in several metabolic pathways, including tryptophan metabolism. The microbiota-derived tryptophan metabolite indole-3-lactic acid can induce intestinal ischemia injury^11^ and sepsis^12^. Hypoxia-induced lactate can modulate the cancer microenvironment to sustain cancer cell survival^13^. Under hypoxic conditions, plasma levels of lactate in mice increase alongside the expression of several inflammatory cytokines, such as IL-6, resulting in kidney injury^14^. Lactate levels are associated with changes in the gut microbiota and may have a critical role in disease regulation. As nano-MPs induce hypoxia and promote oxidative stress, we hypothesized that they may also induce changes in the gut microbiota. In particular, an imbalance among lactic acid bacteria due to the hypoxic environment may lead to excessive lactate generation in the gut, plasma and lungs through the gut–lung axis, with subsequent exacerbation of LDMP by promoting the epithelial–mesenchymal transition (EMT). This process may be ameliorated by gut microbiota depletion and fecal microbiota transplantation (FMT). This study provides a deeper understanding of the potential pathological mechanisms underlying LDMP and offers potential targets for its prevention and control.

## Materials and methods

### Nano-MPs

The nano-MPs used in this study were polystyrene nanoplastics purchased from Xi’an Qiyue Chuanke Biotechnology Co., Ltd. (Shaanxi, China) (2.5% w/v, 10 mL). Their morphologies were determined via transmission electron microscopy (TEM). The size distribution and zeta potential were also determined using a dynamic light-scattering device (Zetasizer; Malvern Instruments, Malvern, Worcestershire, UK). The samples were stored in our laboratory at 2–8 °C until use in experiments.

### Animals and experimental design

C57BL/6 male mice (8 weeks old, 18 ± 2.5 g) were purchased from Hunan SJA Lab Animal Co., Ltd. (Hunan, China) and raised at the Animal Lab Division, Xiangya School of Public Health, Central South University, China. Animal rearing and interventions were conducted per the guidelines of the Lab Animals Center at Central South University and Chinese national regulations for alleviating suffering. The study was approved by the Institutional Animal Review Board of Central South University (approval No. 2020sydw0110). The mice were maintained in a specific-pathogen-free environment under controlled conditions (humidity: 55%±5%; temperature: 21 °C ± 2 °C; 12/12-h light/dark cycle) with access to chow and water *ad libitum*. The mice could acclimate to the laboratory environment for 1 week before the experiments.

The study involved five animal experiments: nano-MP exposure, antibiotic cocktail intervention, FMT, lactate intervention, and *PTBP1* knockout combined with lactate.

In exposure experiments, the mice were divided into a control group (Con) and a treatment group (nano-MPs) (*n* = 20 per group). Mice in the latter group received intratracheal administration of 15 mg/kg nano-MPs three times per week, while controls received deionized water via the same route and schedule. The mice were sacrificed 28 days after the first dose, and lung tissues were dissected for analysis.

In the antibiotic cocktail intervention experiment, the mice were divided into the following groups: control (Con), antibiotic cocktail treatment to deplete the gut microbiota (Cocktail), nano-MP treatment (nano-MPs), and antibiotic cocktail plus nano-MP treatment (Cocktail+Nano-MPs) (*n* = 15 per group). The antibiotic cocktail consisted of 50 g/L ampicillin sodium salt (CA2031–5 G), 50 mg/L polymyxin B sulfate salt (CP8711–100 MG), and 100 U/L streptomycin sulfate (CS 10481–5 G), all of which were purchased from the China Center of Industrial Culture Collection (http://www.china-cicc.org). The concentration of nano-MPs was the same as that in the nano-MP exposure experiment. The antibiotic cocktail was suspended in saline and administered at 100 μL per mouse by oral gavage once per day for 1 week.

The FMT experiment was performed as described previously. Briefly, 10 male donor mice were divided into a nano-MP donor group gavaged with nano-MPs as described above and a control donor group gavaged with the same volume of phosphate buffered saline (PBS). Fresh fecal samples were harvested in sterile saline, mixed, suspended, and centrifuged, and the supernatants were collected for transplantation. Specific-pathogen-free mice were transplanted intragastrically with feces harvested from either nano-MP or control donors for 1 week.

In the lactate intervention experiment, mice were divided into vehicle, lactate, nano-MP, and lactate plus nano-MP treatment (Lactate+Nano-MP) groups (*n* = 10 per group). Mice in the lactate groups received an intraperitoneal (i.p.) injection of 5 mM/kg/d lactate for 28 days, while the nano-MP treatment groups were treated with nano-MPs as described above.

Finally, the mice were divided into the following groups: nano-MP intervention in *Ptbp1*-wt mice (WT+Nano-MP group), lactate combined with nano-MP intervention in *Ptbp1*-wt mice (WT+Lactate+Nano-MP group), nano-MP intervention in *Ptbp1*-knockout mice (*Ptbp1*^–/–^+Nano-MP group), and lactate and nano-MP intervention in *Ptbp1*-knockout mice (*Ptbp1*^–/–^+Lactate+Nano-MP group) (*n* = 10 per group). The lactate groups received i.p. injections of 5 mM/kg/d lactate for 28 days, while the nano-MP treatment groups were treated with nano-MPs as described above. The *Ptbp1*^–/–^ mice were produced by Cyagen Biosciences (Number: CKOAI210729XY2-B; Santa Clara, CA, USA). Briefly, tissue-specific gene deletion was confirmed by adding one additional primer to the PCR assay:

F1: 5′-CAGGGTTGGTTCGGCTAAAATAAT-3′

R2: 5′-AAGACTGAGTGATTAGAGGCTTGTT-3′.

### Cell culture and *PTBP1*-knockout cell construction

HBE cells purchased from the American Type Culture Collection (ATCC, Manassas, VA, USA) were cultured in Dulbecco’s modified Eagle’s medium (DMEM; HyClone, Logan, UT, USA) supplemented with 10% fetal bovine serum (FBS) and 1% antibiotics in a 5% CO_2_ incubator at 37 °C. *PTBP1*-knockout (*PTBP1*-KO) HBE cells were generated using a CRISPR/Cas9-based technique (Hesheng Biotech, Co. Ltd., Shanghai, China).

### Antibodies and constructs

We used the following antibodies: anti-PTBP1 (1:1000 for Western blotting, 101043-T46; Sino Biology, China), anti-N-cadherin (1:1000 for Western blotting and 1:200 for immunofluorescence analysis, 13116 s; Cell Signaling Technology, Danvers, MA, USA), anti-TGF-β (1:1000 for Western blotting and 1:200 for immunofluorescence analysis, #3711; Cell Signaling Technology), and anti-GAPDH (1:1000 for Western blotting and 1:200 for immunofluorescence analysis, TA-08; ZSGB-BIO, Beijing, China).

### Immunofluorescence analysis

Immunofluorescence analysis was performed as described previously. The lung tissues were fixed with 4% paraformaldehyde and cut into frozen sections. The sections were cleared with 0.3% Triton X-100 and blocked with 2% bovine serum albumin (BSA). Then, they were washed with PBS, incubated with the primary antibody, and treated with an appropriate secondary antibody. Next, they were incubated in the dark at room temperature for 1 h. After washing with PBS, the nuclei were stained with 4′,6-diamidino-2-phenylindole (DAPI; Sigma‒Aldrich, St. Louis, MO, USA), and the sections were mounted and observed via confocal microscopy (X-Light V3; CrestOptics, Rome, Italy)^15^.

### Western blotting analysis

Lung tissue samples or cultured cells were lysed in cell lysis buffer to extract the total protein with constant agitation for 30 min at 4 °C. Nuclear and cytoplasmic proteins were isolated using a Nuclear and Cytoplasmic Protein Extraction Kit (Beyotime Biotechnology, Shanghai, China). Western blotting analysis was performed as described previously. Briefly, the proteins were separated via sodium dodecyl sulfate (SDS)-polyacrylamide gel electrophoresis (PAGE), transferred onto polyvinylidene fluoride (PVDF) membranes (Roche, Mannheim, Germany) using a Mini-Trans Blot (Bio-Rad, Hercules, CA, USA), and labeled through incubation with the appropriate antibodies as described above. Signals were visualized using an ImageQuant LAS4010 (GE Healthcare Biosciences, Piscataway, NJ, USA). Protein gray analysis was performed using ImageJ software^16^.

### Quantitative real-time polymerase chain reaction (PCR)

Quantitative real-time PCR (qRT‒PCR) was performed as described previously. RNA was extracted from cells or tissues using TRIzol reagent (Jingcai Bio, Xi’an, Shaanxi, China). Quantification was performed using SYBR green dye (TB Green Premix Ex Taq II; Takara Bio, Shiga, Japan). The PCR profile consisted of an initial denaturation step at 95 °C for 10 min, followed by 40 cycles of 95 °C for 15 s and 60 °C for 1 min. Detection was performed using a CFX96 Touch System (Bio-Rad), and relative expression was calculated using the 2^–ΔΔCT^ method.

The primers used were as follows:

E-cadherin forward: 5′-GCCTCCTGAAAAGAGAGTGGAAG-3′,

E-cadherin reverse: 5′-TGGCAGTGTCTCTCCAAATCCG-3′;

N-cadherin forward: 5′-CCTCCAGAGTTTACTGCCATGAC-3′;

N-cadherin reverse: 5′-GTAGGATCTCCGCCACTGATTC-3′;

Vimentin forward: 5′-AGGCAAAGCAGGAGTCCACTGA-3′;

Vimentin reverse: 5′ -ATCTGGCGTTCCAGGGACTCAT-3′.

### Hematoxylin & eosin and Masson’s trichrome staining

Fixed lung tissues were dehydrated, embedded in paraffin, and cut into 4-μm-thick sections. After deparaffinization and dehydration, the sections were stained with hematoxylin & eosin (H&E). The sections were also stained with Masson’s trichrome according to the manufacturer’s instructions (DC0033; Leagene Biotechnology, Beijing, China).

### Fluorescence in situ hybridization

The cells were fixed, permeabilized, and incubated with primary antibodies against two different proteins, followed by secondary antibodies conjugated with different fluorophores. The cells were imaged via confocal microscopy, and the colocalization of two proteins was observed as overlapping signals in merged images. The signals were detected using the Fluorescent In Situ Hybridization Kit (RiboBio, Guangzhou, China) following the manufacturer’s instructions. Images were taken using an immunofluorescence confocal microscope (X-Light V3; CrestOptics).

### Microbial analysis

The V3–V4 regions of the 16 S rRNA gene were amplified and sequenced using the Illumina HiSeq2500 sequencing platform (Illumina, San Diego, CA, USA). The raw sequence data were quality-controlled using QIIME (v1.9.1) with the default parameters and then demultiplexed and clustered into 50 taxonomic units at the species level (97% similarity). Operational taxonomic unit generation was based on the Greengenes database (v13_8) and the reference-based method from SortMeRNA. Strain composition, alpha diversity, and beta diversity analyses were performed using QIIME (v1.9.1)^17^.

### Lactate detection

Lactate levels were determined using the CheKine Micro Lactate Assay Kit (KTB1100; Abbkine, Wuhan, China).

### Statistical analysis

All data are reported as the mean ± standard deviation. Differential mRNA expression was defined as a fold change ≥ 2.0 and *p* < 0.05. Unpaired numerical data were compared using the unpaired *t*-test (two groups) or analysis of variance (more than two groups). Statistical analyses were performed using SPSS for Windows (ver. 22.0; SPSS Inc., Chicago, IL, USA), and the data were plotted using GraphPad Prism 9 (GraphPad Software, San Diego, CA, USA). In all of the analyses, *p* < 0.05 was taken to indicate statistical significance.

## Results

### Exposure to nano-MPs induces lung and intestinal damage simultaneously

**Fig. 1:**
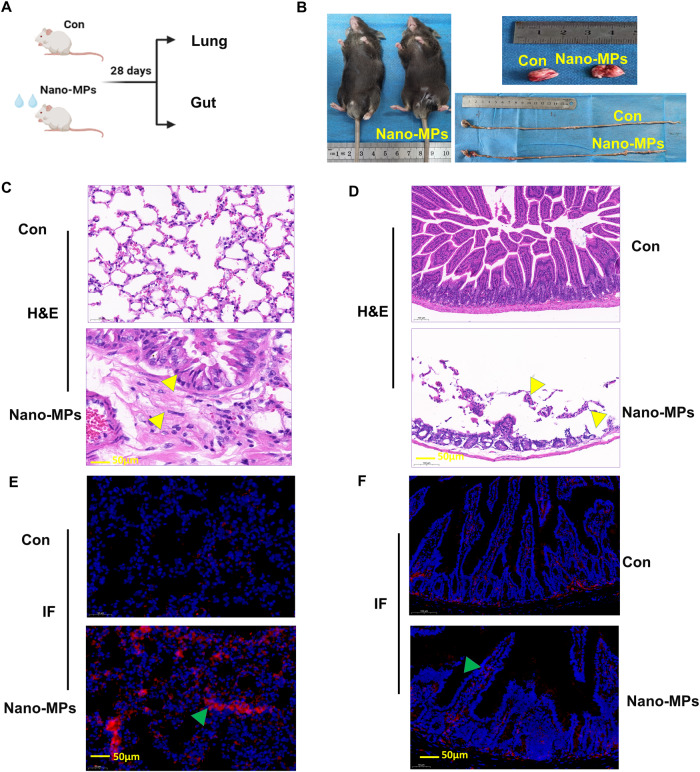
Short-term exposure to a large amount of nano-MPs caused lung and intestinal damage. **A** Design of the mouse experiments. C57BL/6 J mice were divided into two groups: controls (Con) and those treated with nano-MPs (Nano-MP group) (*n* = 20 in each group). In the Nano-MP group, mice received 15 mg/kg nano-MPs via intratracheal administration. Mice in the control group were given deionized water via the same route. All of the mice were treated three times in 1 week. The mice were sacrificed 28 days after the first dose to obtain lung tissues. **B** Photos of lung tissues and intestinal tissues. **C** Images of H&E staining of lung tissues. **D** Images of H&E staining of intestinal tissues. **E** Images of N-cadherin expression in lung tissues via IF detection. **F** Images of N-cadherin expression in intestinal tissues. *n* = 5 in each group for (**B**–**F**); all scale bars: 50 µm.

The nano-MPs used in this study ranged from 50 to 100 nm with a zeta potential of −32.0 and a zeta deviation of 11.6, as determined by transmission electron microscopy (TEM; Supplementary Fig. 1). Compared to the control group, mice treated with nano-MPs showed simultaneous damage in the lungs and intestines (Fig. 1A). Mice in the nano-MP group showed swelling and congestion of the lung tissues and shortening of the intestine compared to tissues in controls. After 28 days of exposure, we collected and processed the lungs and intestines of mice for histological analysis. Exposed mice showed extensive structural changes in the lungs with blood immune cell infiltration, interstitial edema, and thickening of the pulmonary stroma compared to those in controls. They also showed extensive structural changes in the intestine, with intestinal mucosa barrier dysfunction and reduced mucosal thickness and villus density (Fig. 1B–D). Immunofluorescence analysis of N-cadherin, an epithelial cell EMT biomarker^18^, increased in the lung and intestinal tissues of treated mice (Fig. 1E, F). These results indicate that nano-MPs can simultaneously damage the lungs and intestines.

### Abnormal lactate accumulation in the lungs, intestines, and serum is linked to dysregulation of the gut microbiota following exposure to nano-MPs

**Fig. 2:**
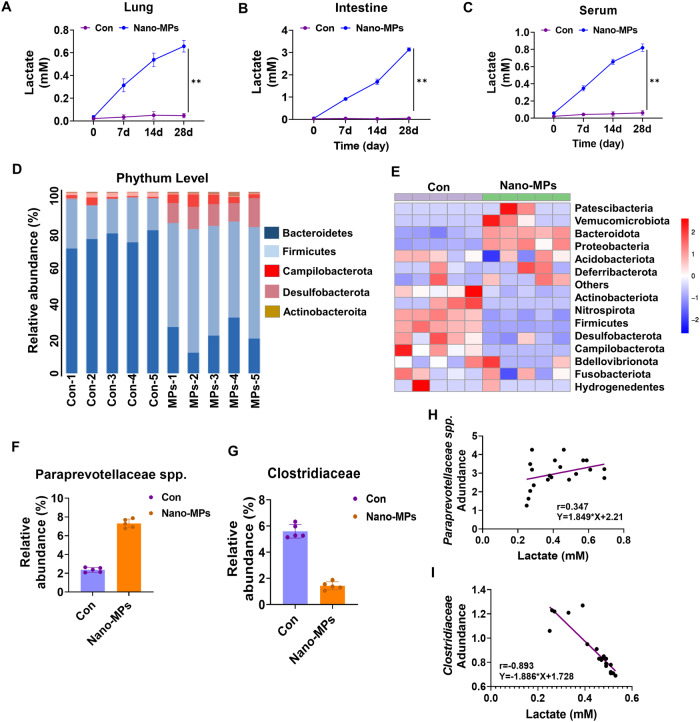
Elevated lactate levels in the lungs are related to gut microbiota dysbiosis. Lactate levels increased in (**A**) lung tissues, (**B**) intestinal tissues, and (**C**) serum after nano-MP treatment. **D** Gut microbiota composition at the phylum level. **E** Cluster analysis of the top 15 most abundant taxa at the phylum level. **F** There was an increased abundance of lactate-producing strains at the genus level (e.g., Paraprevotellaceae) and (**G**) a decrease in the abundance of lactate-consuming strains (e.g., Clostridiaceae). The lactate levels in the lungs were (**H**) positively correlated with Paraprevotellaceae abundance but (**I**) negatively correlated with Clostridiaceae abundance (*n* = 5 in each group). Data are expressed as the means ± SEMs. *p < 0.05; **p < 0.01 vs. the control group. *n* = 5 in each group, unless otherwise stated.

Exposure to plastic particles induces an increase in lactate dehydrogenase activity^19^, which is responsible for converting pyruvate into lactate to maintain glycolysis^20^. We found that exposure to nano-MPs increased lactate levels in the lungs, intestines, and serum (Fig. 2). The levels peaked after 28 days of treatment. As changes in lactic acid levels are closely associated with the gut microbiota^21^, we examined differences in the gut microbiota between the control and treatment groups via 16 S rRNA gene sequencing. At the phylum level, the Firmicutes/Bacteroidetes ratio was increased in the treatment group (Fig. 2); the abundances of Campylobacterota and Desulfobacterota were also higher. Cluster analysis showed enrichment of Patescibacteria, Verrucomicrobiota, and Bacteroidetes in the treatment group. The abundance of Paraprevotellaceae increased, while that of Clostridiaceae decreased after treatment. Moreover, the abundance of Paraprevotellaceae was positively correlated with the lactate level (*r* = 0.347, *P* < 0.01), while that of Clostridiaceae was negatively related to the lactate level. These results indicate that exposure to nano-MPs affected the regulation of lactate production and the composition of the gut microbiota.

**Fig. 3:**
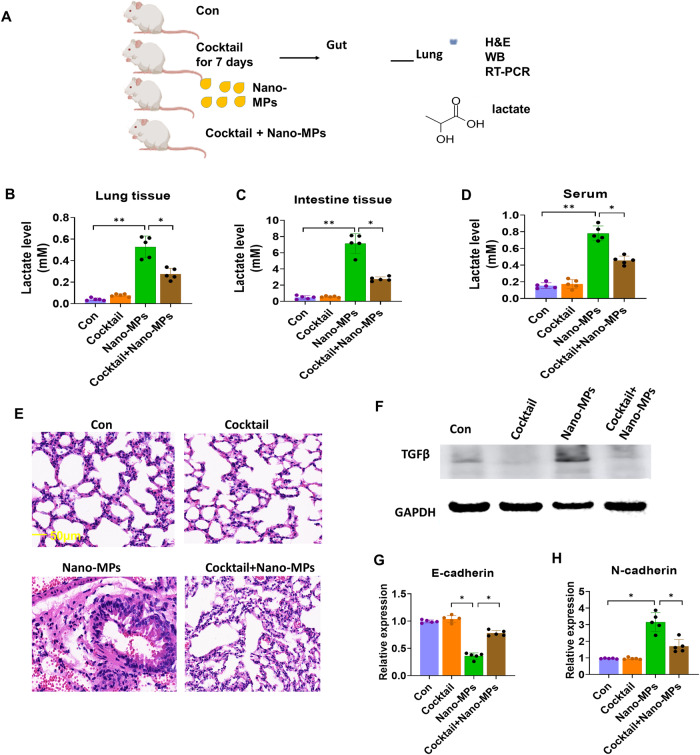
The antibiotic cocktail reversed the lung damage induced by nano-MPs and decreased lactate levels in lung tissues. **A** Design of the mouse experiments. C57BL/6 J mice were divided into four groups: controls (Con), those in which the gut microbiota was depleted via antibiotic cocktail treatment (Cocktail), mice treated with nano-MPs (Nano-MP group), and mice treated with a combination of the last two treatments (Cocktail+Nano-MP group) (*n* = 15 in each group). The antibiotics reversed the increased lactate levels in the (**B**) lungs, (**C**) intestines, and (**D**) serum (all, *n* = 5). **E** H&E staining images (bar = 50 μm). The antibiotics reversed (**F**) TGF-β expression (*n* = 3), (**G**) E-cadherin mRNA expression (*n* = 5), and (**h**) N-cadherin mRNA expression (*n* = 5) in lung tissues. Data are expressed as the means ± SEMs. *p < 0.05; **p < 0.01 vs. the control group.

We hypothesized that the increased lactate levels in lung tissues following exposure to nano-MPs might be derived from the gut microbiota because higher proportions of lactate-producing genera such as *Lactobacillus*, *Streptococcus*, and *Turicibacter* are positively correlated with disease, including hypertension^22^ and liver fibrosis^23^; additionally, lactate levels are significantly correlated with the abundances of Firmicutes, Bacteroidetes, and Proteobacteria, which are associated with advanced fibrosis^23^. Mice were orally administered a cocktail of antibiotics to deplete the gut microbiota (Fig. 3). This depletion led to a decrease in lactate levels in the lungs, intestines, and serum after treatment with nano-MPs. Moreover, histological analysis showed reduced lung damage in the group treated with the antibiotic cocktail. The expression of transforming growth factor beta (TGF-β) was decreased, indicating amelioration of the inflammation induced by exposure to nano-MPs. Exposed mice subsequently treated with the antibiotic cocktail showed increased E-cadherin expression and decreased N-cadherin expression. These results suggest that increased lactate levels in the lungs following exposure to nano-MPs may be derived from the gut microbiota.

### LDMP is transmissible via the gut microbiota, partially through lactate

**Fig. 4:**
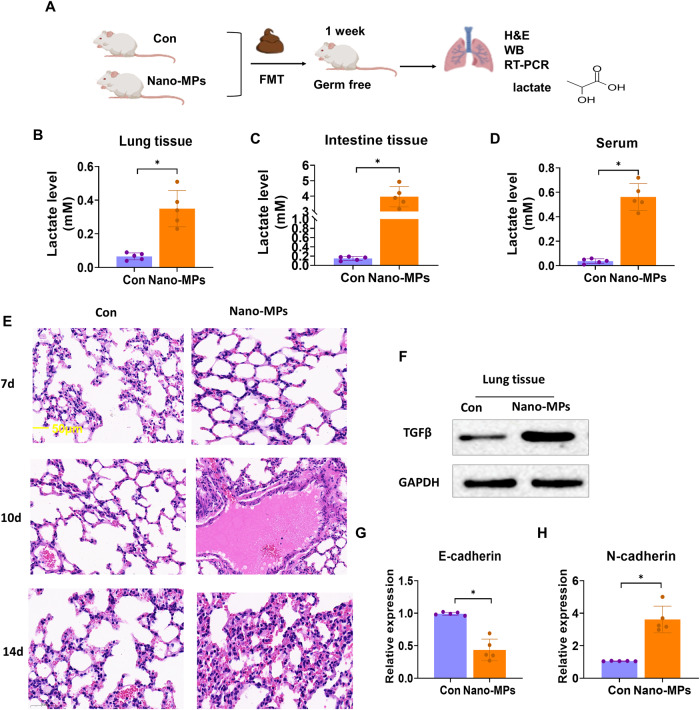
FMT from conventional donor mice treated with nano-MPs transplanted into germ-free mice aggravates lung damage and increases lactate levels. **A** Germ-free mice were transplanted with feces harvested from conventional mice undergoing a sham operation and the nano-MP operation intragastrically for 1 week. **B** Lactate levels in lung tissues were increased in the Nano-MP group compared to those in the control group. *n* = 5. **C** Lactate levels in intestinal tissues were increased in the Nano-MP group compared to the control group. *n* = 5. **D** Lactate levels in serum were increased in the Nano-MP group compared to the control group. *n* = 5. **E** Representative H&E staining images (bar = 50 μm). **F** TGF-β protein expression in lung tissues was increased in the Nano-MP group compared to the control group. *n* = 3. **G** E-Cadherin mRNA expression in lung tissues was decreased in the Nano-MP group compared to the control group. *n* = 5. **H** N-Cadherin mRNA expression in lung tissues was increased in the Nano-MP group compared to the control group.

FMT was performed to examine the gut microbiota-mediated phenotype of LDMP and the elevation of lactate levels (Fig. 4). Germ-free mice that underwent FMT with samples from mice treated with nano-MPs showed elevated levels of lactate in the lungs, intestines, and serum compared to levels in mice that received samples from sham-treated control mice. Moreover, their lung tissues showed a greater degree of damage in a time-dependent manner. In addition, they had higher expression levels of TGF-β, indicating exacerbated inflammation, decreased E-cadherin expression, and increased N-cadherin expression. These results suggest that the increased lactate levels due to exposure to nano-MPs may be mediated by changes in the gut microbiota, leading to exacerbated lung damage through the gut–lung axis.

### Lactate exacerbates LDMP through EMT

**Fig. 5:**
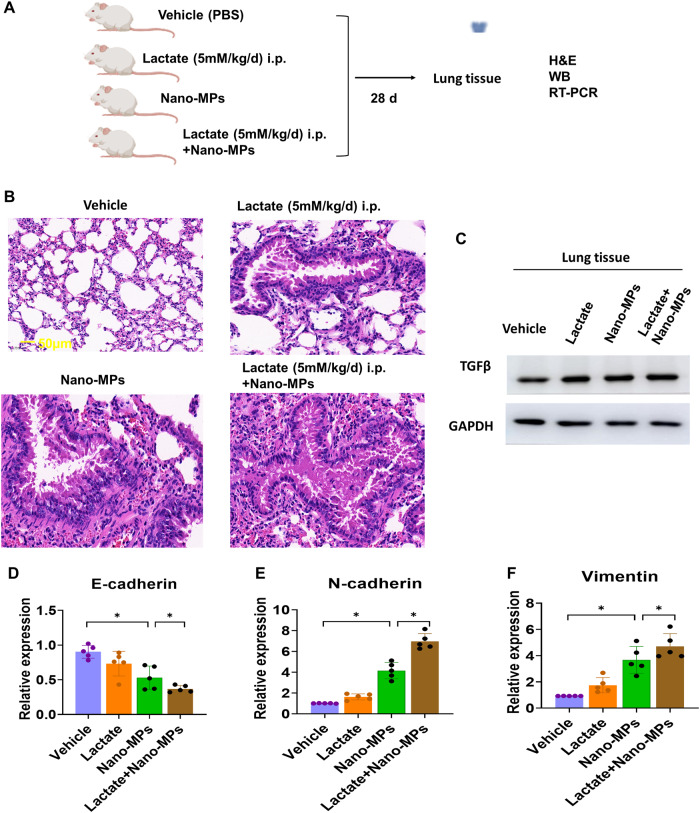
Lactate aggravated nano-MP-induced lung damage. **A** C57BL/6 J mice were divided into four groups: control (Con), lactate given via intraperitoneal (i.p.) injection of 5 mM/kg/d for 28 days (Lactate group), mice treated with nano-MPs (Nano-MP group), and a combination of the last two groups (Lactate+Nano-MP group) (*n* = 10 in each group). **B** H&E staining images (bar = 50 μm: *n* = 2). Lactate aggravated nano-MP-induced (**C**) TGF-β protein expression (*n* = 3), (**E**) N-cadherin mRNA expression (*n* = 5), and (**F**) vimentin mRNA expression (*n* = 5) but inhibited (**D**) E-cadherin mRNA expression (*n* = 5). Data are expressed as the means ± SEMs. *p < 0.05; **p < 0.01 vs. the control group.

**Fig. 6:**
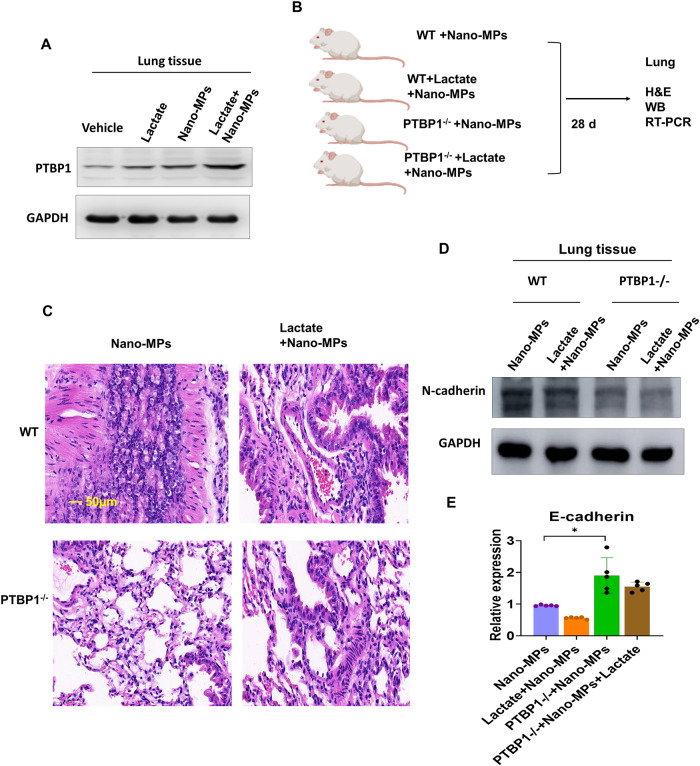
PTBP1 signaling mediated lactate-induced EMT and lung damage after treatment with nano-MPs. **A** Western blotting showed that treatment with lactate combined with nano-MPs increased PTBP1 protein expression. GAPDH was used as a control. **B** C57BL/6 J mice were divided into four groups: PTBP1-wt mice treated with nano-MPs (WT+Nano-MP group), PTBP-wt mice treated with lactate and nano-MPs (WT+Lactate+Nano-MP group), PTBP1^–/–^ mice treated with nano-MPs (PTBP1^–/–^+Nano-MP group), and PTBP1^–/–^ mice treated with lactate and nano-MPs (PTBP1^–/–^+Lactate+Nano-MP group). Lactate was administered at 5 mM/kg/d, and nano-MPs were administered at 15 mg/kg nano-MPs intratracheally for 28 days. **C** H&E staining images (bar = 50 μm: *n* = 2). **D** PTBP1^–/–^ attenuated the nano-MP-induced N-cadherin protein expression (*n* = 3). **E** PTBP1^–/–^ increased the nano-MP-induced E-cadherin protein expression (*n* = 5). Data are expressed as the means ± SEMs. *p < 0.05; **p < 0.01 vs. the control group.

Mice were treated with vehicle as a control or with lactate alone, nano-MPs alone, or lactate plus nano-MPs (Fig. 5). Lung tissues were collected and examined. The lung damage was more severe in mice after exposure to lactate plus nano-MPs, with greater collagen hyperplasia and interstitial thickening than those in the other three groups. TGF-β expression increased in the three treatment groups, indicating that nano-MPs and/or lactate induced inflammation. There was decreased E-cadherin expression and increased N-cadherin and vimentin expression in the lactate plus nano-MP group. PTBP1 plays an essential role in EMT^15^. Knockout of PTBP1 may alleviate radiation-induced EMT. Therefore, we hypothesized that PTBP1 may regulate the EMT process during the development of lung damage in this context. PTBP1 expression was higher after treatment with lactate plus nano-MPs than in the lactate-alone group (Fig. 6). These results suggest that lactate supplementation may exacerbate such lung damage by regulating EMT biomarkers. PTBP1 may be a key regulator of the response.

### PTBP1 is essential for the effect of lactate on LDMP

**Fig. 7:**
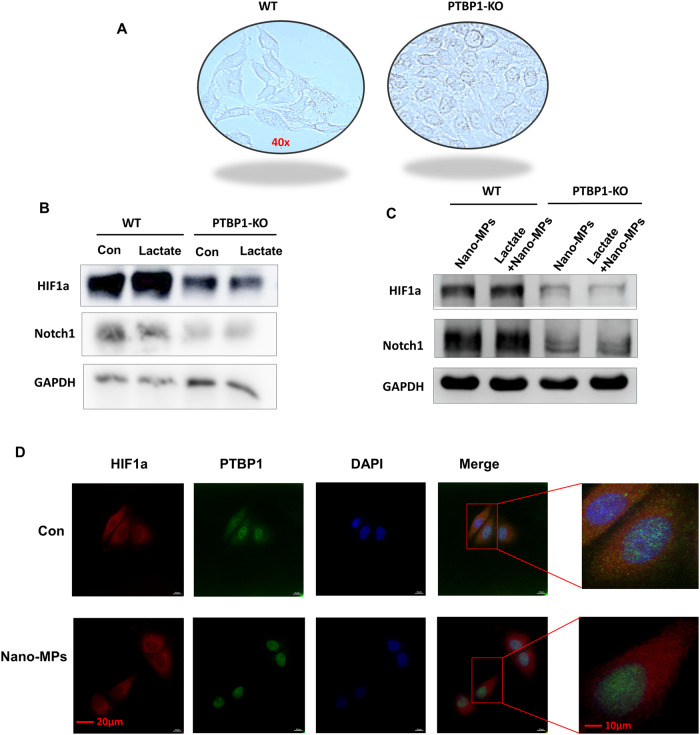
HIF1a/PTBP1 signaling was required for lactate-induced EMT in lung cells. **A** Representative images of the cell phenotypes of PTBP1-wt and PTBP1-KO HBE cells. The magnification is 40×. **B** HIF1a protein expression in the control and lactate-treated groups with or without PTBP1 expression in lung cells. **C** HIF1a protein expression in the control and lactate-treated groups of HBE cells with or without PTBP1 expression. **D** Confocal microscopy of lung cells showed that HIF1a colocalized with PTBP1 in the nucleus after nano-MP treatment. Scale bar = 20 μm.

We generated conditional lung-specific *Ptbp1*-knockout mice (*Ptbp1*^lung–KO^) to explore the mechanism underlying the effects of lactate in LDMP mediated by EMT. Next, we examined the effects of treating wild-type (*Ptbp1*^-wt^) mice and *Ptbp1*^lung–KO^ mice with nano-MPs alone, or lactate plus nano-MPs (Fig. 6). Lung tissues were collected and showed amelioration of lung damage in *Ptbp1*^lung–KO^ mice after exposure to nano-MPs alone or lactate plus nano-MPs. N-Cadherin expression was decreased in *Ptbp1*^lung–KO^ mice after exposure to nano-MPs alone or lactate plus nano-MPs, indicating inhibition of the EMT process under conditions of PTBP1 deficiency. E-Cadherin expression was also increased in *Ptbp1*^lung–KO^ mice after exposure to nano-MPs alone or lactate plus nano-MPs. Next, we constructed a *PTBP1*-knockout (*PTBP1*-KO) cell line in human bronchial epithelial (HBE) cells. As the high lactate environment represents hypoxic conditions^24^, we examined the expression of HIF1a because it regulates EMT^25^. Knockout of *PTBP1* increased the cell growth rate (Fig. 7). HIF1a and Notch1 expression levels were decreased in *PTBP1*-KO cells after lactate treatment and following exposure to nano-MPs alone or lactate plus nano-MPs. A protein in situ localization assay suggested that exposure to nano-MPs induced PTBP1 by activating HIF1a overexpression. These results indicate that nano-MPs induce EMT in the lungs by regulating the HIF1a/PTBP1 signaling pathway and that increased lactate levels exacerbate LDMP by regulating the same pathway. Therefore, the HIF1a/PTBP1 signaling pathway may be a key regulator of the response to LDMP.

## Discussion

Nano-MPs simultaneously induced damage to the lungs and intestines, and gut microbiota-derived lactate exacerbated LDMP by regulating EMT. There was an abnormal increase in lactate levels in the lungs of mice exposed to nano-MPs and close associations with the dysbiosis of lactic acid bacteria and metabolic pathways. The nano-MP-induced elevation of lactate levels was decreased in germ-free mice and reversed by depleting the gut microbiota using an antibiotic cocktail in conventional mice. This finding suggests that lactate secretion was derived from the gut microbiota after treatment with nano-MPs. The HIF1a/PTBP1 signaling pathway mediated the exacerbation of LDMP by lactate. Our results indicate that lactate derived from the gut microbiota can exacerbate LDMP and that the lactate/HIF1a/PTBP1 signaling pathway plays a role in this reaction.

Excessive lactate accumulation is a common metabolic event under hypoxic conditions. It is involved in cancer cell proliferation^26^, hepatic gluconeogenesis^27^, acidosis, and muscle pain^28^. It may lead to an acidic cancer microenvironment, promoting progression^29^. In addition, in the nucleus, it may decrease histone deacetylase expression and increase histone acetylation^26^ or induce cancer cell immune evasion^30^. Our results demonstrate abnormal lactate accumulation in the lungs, intestines, and plasma after exposure to nano-MPs, leading to tissue damage. Interestingly, polystyrene nanoplastics can activate HIF1a expression to form a hypoxic environment, impair testosterone synthesis and male reproductive function^31^, and induce apoptosis and necroptosis in swine testis cells^32^. Because lactate levels increase under hypoxia^33^, there is a metabolic shift toward glycolysis^34^. This study provides new insights into the role of lactate in LDMP and suggests that it is a potential target for preventive and therapeutic strategies. Furthermore, excessive lactate accumulation in the lungs may be closely associated with changes in the gut microbiota. In addition, the integrity of the intestinal mucosa was severely disrupted by exposure to nano-MPs. Similarly, previous studies have found that polyethylene terephthalate impairs gut immune homeostasis^35^, enhances intestinal permeability^36^, and disrupts the gut microbiota in the carp intestine^37^. We found no significant changes in the abundance of classic lactic acid-producing bacteria, such as *Lactobacillus rhamnosus*. However, there was an imbalance in the Firmicutes/Bacteroidetes ratio in the gut microbiota following exposure to nano-MPs. An imbalance in this ratio is a hallmark of intestinal barrier damage and inflammation induced by a high-fat diet and triggers increased serum levels of lactic acid^38^. Therefore, lactate accumulation following exposure to nano-MPs may be due to the Firmicutes*/*Bacteroidetes ratio imbalance in the gut microbiota. Our evidence suggests that the gut microbiota likely produces lactate and is an essential regulator of LDMP.

The gut–lung axis is affected by airborne pollution, such as particulate matter^39^. Nano-MPs can accumulate in multiple tissues, including the liver, brain, lungs, and gut^40^. Polystyrene nanoplastics can cross the intestinal barrier^5^, and long-term exposure can result in dysfunctional intestinal mucus secretion and intestinal injury^41^. Our study shows that exposure to nano-MPs can concurrently induce lung and intestinal damage.

Lactate promotes EMT induced by nano-MPs. EMT is a common process in cancer metastasis^42^, with a decrease in the E-cadherin epithelial biomarker and an increase in the N-cadherin and vimentin mesenchymal cell biomarkers^43^. Epithelial cells in the lungs undergo EMT in response to nanoplastics, indicating a toxicological effect in the respiratory system^44^. In our study, lactate levels in the serum were elevated after exposure to nano-MPs. Excessive lactate accumulation in the serum may be responsible for the subsequent accumulation in the lungs because such accumulation is associated with lung alveolar damage and activation of the inflammatory factor TGF-β. Treatment with lactate decreased epithelial biomarker expression but increased the expression of mesenchymal biomarkers, suggesting the essential role of EMT in mediating the effects of lactate on LDMP. Therefore, lactate may be a useful circulating biomarker for the identification of LDMP.

We also showed that the HIF1a/PTBP1 signaling pathway is crucial for the lactate-induced promotion of LDMP. HIF1a is a hypoxia-inducible transcription factor, while PTBP1 may regulate posttranscriptional modification and alternative splicing^45^. Elevated PTBP1 levels in lung cancer cells can promote EMT induced by radiation exposure^15^. Fu et al. reported a positive feedback loop in the HIF1a/PTBP1/circRNATDR3/HIF1a axis that facilitates colorectal cancer cell proliferation, migration, and metastasis^46^. Our results are consistent with a previous study that reported the involvement of the HIF1a/PTBP1 signaling pathway in the EMT process and suggested a critical role for lactate in regulating HIF1a/PTBP1 activation. Therefore, we conclude that the effects of lactate on EMT induced by nano-MPs are regulated partly by the HIF1a/PTBP1 pathway. However, HIF1a activation can also induce multiple downstream signaling pathways in response to different stimuli or in different cell lines; further studies are required to identify the signaling pathways involved and the roles of PTBP1 and lactate in the effects of nano-MPs.

This study had some limitations. First, lactate may have been generated from other sources, including other lactic acid bacteria or fermented foods^47^, or the production of secondary metabolites^48^. Therefore, further studies should focus on host-derived sources of lactate. Second, the functions and mechanisms of action of lactate were only analyzed in an animal model and cell culture experiments; future studies should include serum and fecal samples from patients with LDMP. Finally, other essential gut microbiota metabolites, including short-chain fatty acids, should be investigated in gut and lung tissues.

**Fig. 8:**
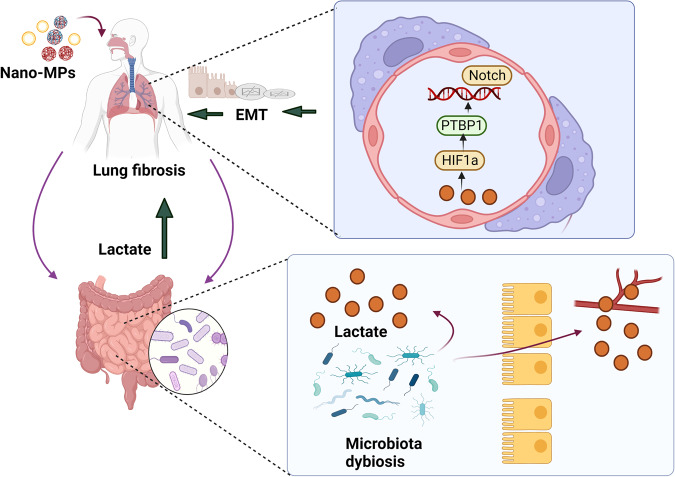
Model of the suggested regulation of nano-MP-induced lung fibrosis. In response to nano-MPs, the gut microbiota composition changed, and the abundance of lactate-producing bacteria increased; moreover, the intestinal barrier was damaged, and the lactate was able to enter the blood circulation and accumulate in the lung. Lactate aggravated nano-MP-induced lung fibrosis by triggering the HIF1a/PTBP1 signaling pathway, which is a key regulatory pathway that activates the EMT process, eventually leading to lung fibrosis.

In summary, elevated lactate levels in the lungs following exposure to nano-MPs are derived from the gut microbiota. Mechanistically, the elevated levels trigger the HIF1a/PTBP1 pathway, exacerbating LDMP by modulating the EMT process (Fig. 8). These findings identify lactate as a potential target for the treatment and prevention of LDMP.

### Supplementary information

Supplementary file

## Data Availability

All data needed to evaluate the conclusions in the paper are presented in the paper and/or the Supplementary Materials. Additional data related to this paper may be requested from the authors.
