# Supplementary material for: Lactate exacerbates lung damage induced by nanomicroplastic through the gut microbiota–HIF1a/PTBP1 pathway
Source: Exp Mol Med. 2023 Dec 1;55(12):2596–607. doi: 10.1038/s12276-023-01129-3 (PMC10766629; doi:10.1038/s12276-023-01129-3)
Supplement: Supplementary file 1 — Supplementary file [file 12276_2023_1129_MOESM1_ESM.pdf]

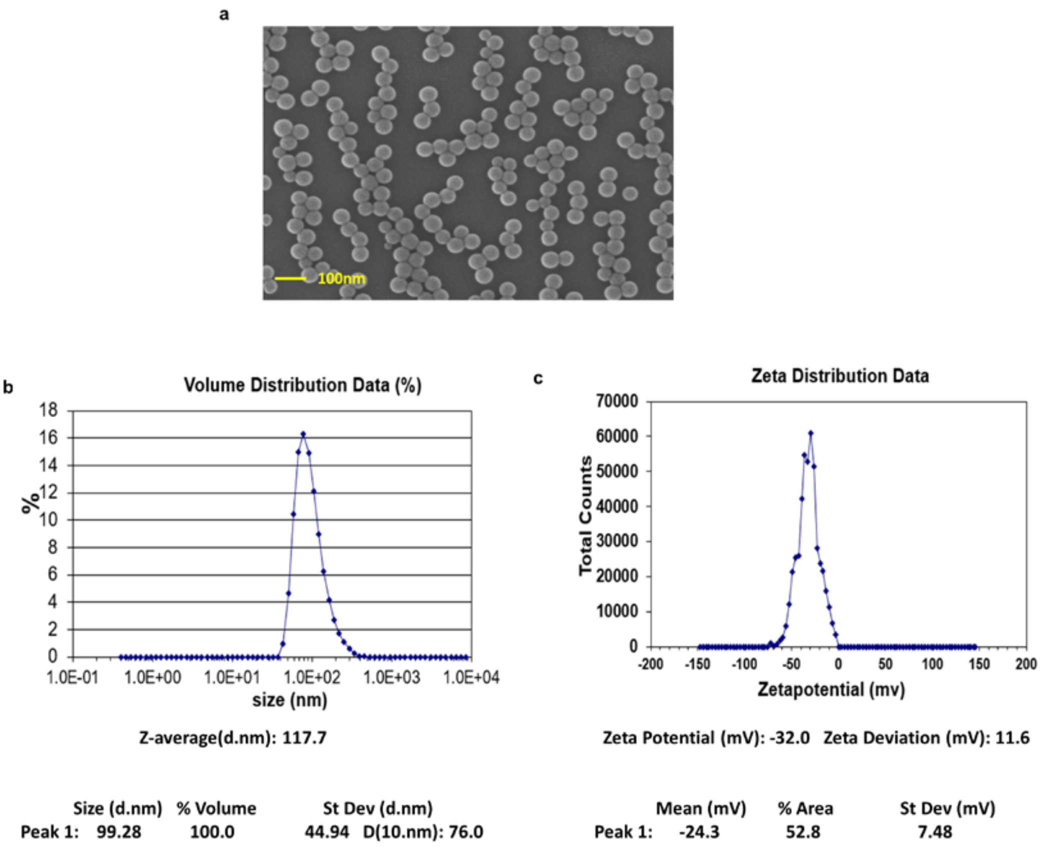

Supplementary Fig. 1 SEM scanning electron microscope pictures of PS-NPs (a), particle size (b), and zeta potential (c).
